# Sphingosine-1-Phosphate and Its Signal Modulators Alleviate Psoriasis-Like Dermatitis: Preclinical and Clinical Evidence and Possible Mechanisms

**DOI:** 10.3389/fimmu.2021.759276

**Published:** 2021-12-21

**Authors:** Liu Liu, Jiao Wang, Hong-jin Li, Shuo Zhang, Meng-zhu Jin, Si-ting Chen, Xiao-ying Sun, Ya-qiong Zhou, Yi Lu, Dan Yang, Ying Luo, Yi Ru, Bin Li, Xin Li

**Affiliations:** ^1^ Department of Dermatology, Yueyang Hospital of Integrated Traditional Chinese and Western Medicine, Shanghai University of Traditional Chinese Medicine, Shanghai, China; ^2^ Shanghai University of Traditional Chinese Medicine, Shanghai, China; ^3^ Institute of Dermatology, Shanghai Academy of Traditional Chinese Medicine, Shanghai, China; ^4^ Department of Dermatology, The First Hospital of Jiaxing and The Affiliated Hospital of Jiaxing University, Jiaxing, China; ^5^ Department of Dermatology, Shanghai Skin Disease Hospital, Shanghai, China

**Keywords:** preclinical evidence, psoriasis, S1P receptor (S1PR) agonists, sphingosine-1-phosphate, sphingosine-1-phosphate signaling modulators, sphingosine kinase 2 inhibitors, systematic review and meta-analysis

## Abstract

**Background:**

Psoriasis is an autoimmune skin disease associated with lipid metabolism. Sphingosine-1-phosphate (S1P) is a bioactive lipid that plays a key role in the development of autoimmune diseases. However, there is currently a lack of comprehensive evidence of the effectiveness of S1P on psoriasis.

**Objective:**

To assess the efficacy and possible mechanism of S1P and its signal modulators in the treatment of psoriasis-like dermatitis.

**Methods:**

Six databases were searched through May 8, 2021, for studies reporting S1P and its signal modulators. Two reviewers independently extracted information from the enrolled studies. Methodological quality was assessed using SYRCLE’s risk of bias tool. RevMan 5.3 software was used to analyze the data. For clinical studies, the Psoriasis Area and Severity Index score were the main outcomes. For preclinical studies, we clarified the role of S1P and its regulators in psoriasis in terms of phenotype and mechanism.

**Results:**

One randomized double-blind placebo-controlled trial and nine animal studies were included in this study. The pooled results showed that compared with control treatment, S1P receptor agonists [mean difference (MD): −6.80; 95% confidence interval (CI): −8.23 to −5.38; p<0.00001], and sphingosine kinase 2 inhibitors (MD: −0.95; 95% CI: −1.26 to −0.65; p<0.00001) alleviated psoriasis-like dermatitis in mice. The mechanism of S1P receptor agonists in treating psoriasis might be related to a decrease in the number of white blood cells, topical lymph node weight, interleukin-23 mRNA levels, and percentage of CD3^+^ T cells (p<0.05). Sphingosine kinase 2 inhibitors ameliorated psoriasis in mice, possibly by reducing spleen weight and cell numbers (p<0.05).

**Conclusions:**

S1P receptor agonists and sphingosine kinase 2 inhibitors could be potential methods for treating psoriasis by decreasing immune responses and inflammatory factors.

## Introduction

Psoriasis is characterized by demarcated erythema covered with silvery-white scales and often accompanied by varying degrees of itching (1). The global prevalence of psoriasis reportedly varies from 0% to 2.1% in children and 0.91% to 8.5% in adults (2). In addition to the high prevalence of psoriasis, comorbidities such as metabolic syndrome (3), hyperuricemia (4), chronic obstructive pulmonary disease (5), and cardiovascular disease (6) cause great burdens for patients with psoriasis. Psoriasis is considered an autoimmune skin disease, and the classic pathogenesis involves an interleukin-17/interleukin-23 (IL-17/IL-23) immune axis disorder. Therefore, biological agents targeting the IL-17/IL-23 signaling axis have been developed, including IL-17 inhibitors, interleukin 12 (IL-12)/IL-23 inhibitors, and tumor necrosis factor-alpha (TNF-α) antagonists (7). Compared with traditional therapies, although biological agents have good curative effects, the risk of recurrence is increased. A recently published meta-analysis showed that anti-IL-23 drugs are likely to cause a series of immunological and non-immunological adverse events, while their long-term use may cause mental illness (8). Therefore, dermatologists are always seeking better treatment options.

Since several studies have reported that patients with psoriasis have comorbid dysfunctional lipid metabolism (9, 10), dermatologists have developed great interest in studying lipid metabolism in patients with psoriasis. S1P is a bioactive lipid produced by the metabolism of sphingolipids and generated by another sphingolipid metabolite, ceramidase (11, 12). Extracellular S1P is recognized by five G protein-coupled receptors, S1P receptors (S1PRs) 1–5, to regulate various biological processes such as cell growth, differentiation, survival, and vascular and epithelial integrity. S1P is synthesized by two sphingosine kinase subtypes 1 and 2 (SPHK1 and SPHK2) and cleaved by S1P lyase, S1P phosphatase 1, and S1P phosphatase 2 (13). S1P signaling reportedly plays a pivotal role in the development of autoimmune diseases. In 2010, two phase III clinical studies published in *The New England Journal of Medicine* reported that fingolimod, an S1PR agonist, was more effective than interferon in the treatment of multiple sclerosis and reduced the recurrence rate by more than 50% (14). In 2018, it became the only multiple sclerosis drug approved to treat children. In the brains of patients with multiple sclerosis, S1PR1 and SIPR3 levels are significantly increased (15); in patients with psoriasis, the serum S1P concentration is also significantly increased (16). A phase II trial showed that ponesimod, a drug targeting S1P signaling, has therapeutic potential for psoriasis (17). Compared with the classical treatment of psoriasis, both immunosuppressants and biologics can aggravate metabolic disorders in patients with psoriasis, and S1P signaling pathway modulators may be a new treatment for psoriasis (18). However, the efficacy and mechanisms of S1P and its signaling modulators in psoriasis have not been fully elucidated. Hence, here we reviewed previous literature, including clinical and preclinical studies on treatment of psoriasis with S1P and its signaling modulators and quantitatively analyzed their therapeutic effects. Furthermore, we quantitatively and qualitatively analyzed the possible therapeutic targets in terms of phenotype and mechanism.

## Methods

### Literature Search Strategy

The PubMed, Embase, Cochrane, Chinese National Knowledge Infrastructure (CNKI), China Biology Medicine disc (CBMdisc), and Wan Fang databases were electronically searched for relevant articles published through May 8, 2021. A combination of subjects and free words was used for the search. Studies reporting the role of S1P or S1P signaling modulators in animal models of psoriasis were identified. The search terms included “psoriasis”, “S1P”, “sphingosine-1-phosphate”, “fingolimod”, and “animal”. These terms were also translated into Chinese and used to search the Chinese databases. The OpenGery database (http://www.opengrey.eu/), Chinese Clinical Trial Registry (http://www.chictr.org.cn/), and ClinicalTrials.gov (https://clinicaltrials.gov/) databases were also searched. This review is conducted in line with the Preferred Reporting Items for Systematic Reviews and Meta-Analysis (PRISMA) guidelines (19).

### Study Selection

Any studies that met the following criteria were included in this systematic review: (1) randomized controlled trials comparing S1P or S1P signaling modulators with any other drugs; (2) psoriasis animal model; and (3) interventions including S1P or S1P signaling modulators. The control conditions were the same amount of non-functional liquid or no treatment. The exclusion criteria were as follows: (1) reviews, case reports, and observational studies; (2) studies without an *in vivo* model; (3) studies without a control group; and (4) duplicate articles. Two reviewers (H.-J.L. and Y.-Q.Z.) independently screened the titles and abstracts according to the inclusion and exclusion criteria. Any differences in opinions were resolved through discussion.

### Data Extraction

Two authors (S.Z. and M.-Z.J.) independently extracted the following information: (1) first author and publication year; (2) animal species, age (weeks), sex; (3) type of psoriasis model; (4) type of intervention, including different S1P-related drugs, control drugs, and administration route; and (5) main outcome measures. Data are presented as mean ± standard deviation. If the data were not provided in the study, we attempted to contact the authors for the original data or used the publicly available WebPlotDigitizer 4.2 (20) (https://automeris.io/WebPlotDigitizer) to extract the data (21).

### Quality Assessment

SYRCLE’s risk of bias tool for animal studies, which was developed by the Systematic Review Centre for Laboratory Animal Experimentation and based on the Cochrane Collaboration risk of bias tool, was used to conduct the quality assessment (22). The SYRCLE tool covers selection bias, performance bias, detection bias, attrition bias, reporting bias, and other sources of bias. Each term was divided into three grades: a judgment of “Yes” implies low risk, a judgment of “No” implies high risk, and an unclear risk implies insufficient details to assess the risk of bias (22). Two reviewers (L.L. and J.W.) independently assessed the risk of bias. In cases of disagreement, a third reviewer (X.L.) joined the discussion to reach consensus.

### Statistical Analysis

RevMan software (version 5.3) was used to analyze continuous data using the mean difference (MD) and 95% confidence intervals (CIs). The I^2^ statistic was used to assess the interstudy heterogeneity. A random-effects model was used if the I^2^ was >50%, and a subgroup analysis was performed to avoid heterogeneity.

## Results

### Characteristics of the Included Studies

**Figure 1:**
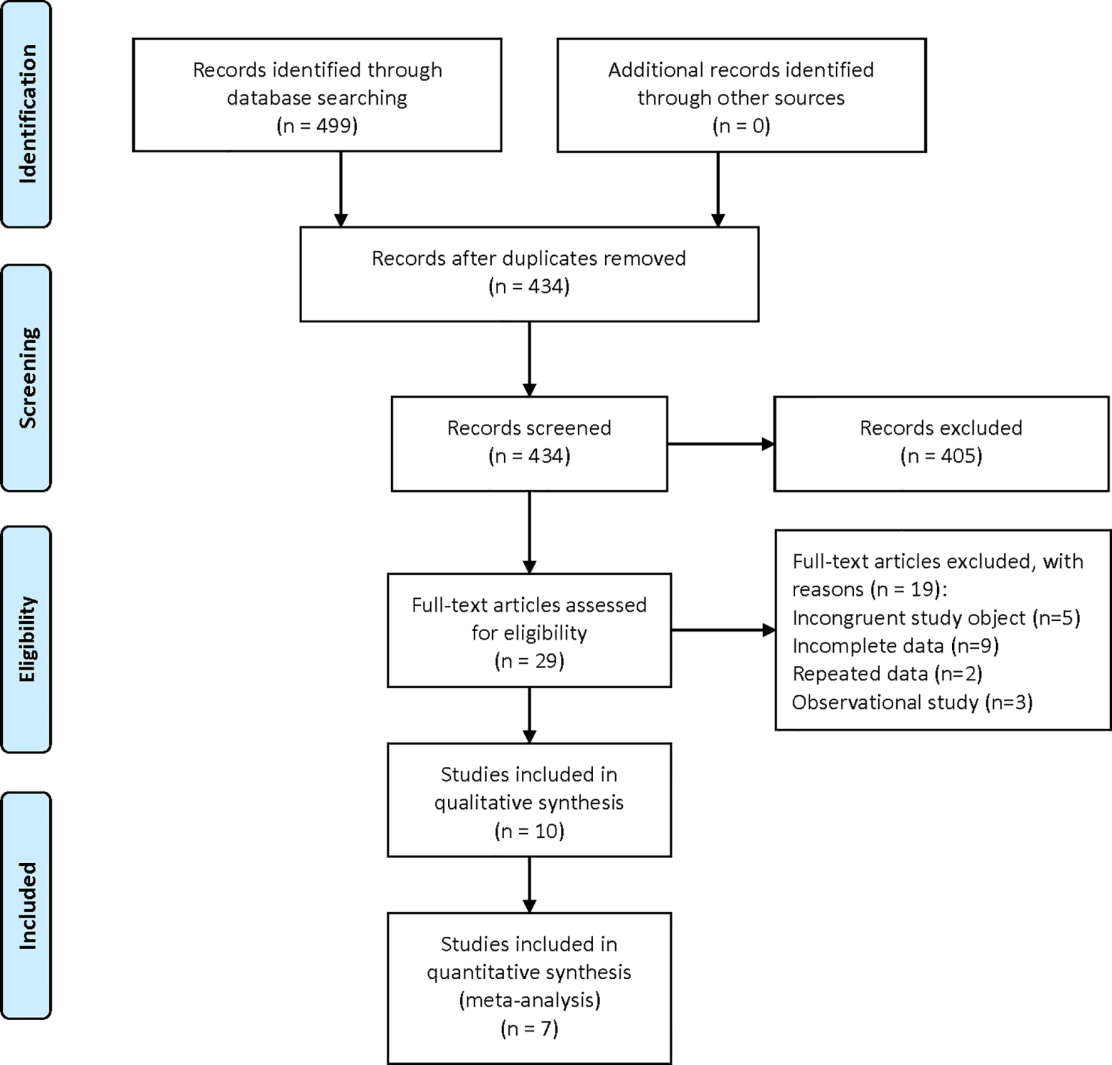
Flowchart of study inclusion according to the PRISMA 2009 guidelines.

A total of 499 relevant studies were identified in the six databases. After the removal of duplicates, 434 articles were subjected to title and abstract screening, which eliminated 405. The remaining 29 studies were subjected to full-text review, which eliminated 17 for having irrelevant research objectives or including incomplete information. Finally, 10 (17, 23–31) studies, including one clinical study and nine preclinical studies, were eligible for inclusion in the qualitative analysis (**Figure 1**).

The characteristics of the included studies are listed in **Table 1** and **Supplementary Table 1**. The clinical study, a randomized double-blind placebo-controlled phase 2 trial, investigated the efficacy and doses of oral ponesimod in the treatment of patients with chronic plaque psoriasis (17). For preclinical studies, four types of experimental mice were used in all studies; BALB/c, C57BL/6, Kunming, and Guinea pigs. Two studies (27, 30) used male mice, one study did not specify the sex of the mice (28), and the fourth study used female mice. Three modeling methods have been used to create psoriasis-like dermatitis, including imiquimod (23, 24, 26–31), propranolol (25, 29) and diethylstilbestrol (25) modeling methods. The interventions consisted of S1P, FTY720 (fingolimod), Syl930, IMMH002, S1P lyase-specific inhibitor (SLI), Ceranib-2, MP-A08, PF-543, ABC294640, and HWG-35D.

**Table 1:** Characteristics of the included preclinical studies.

Study Author and Year	Drug(s)	Species	Sex/Age/Weight	Psoriasis Model	Intervention Group	Control Group	Administration Route	Outcomes
Schaper et al., 2013 (23)	FTY720	BALB/c	Female/6–8 w	50 mg, 5% IMQ	1. IMQ+FTY720	IMQ+vehicle	Intraperitoneal	HE (ET, PCNA), ICI, EE, IL-17, IL-23, LNW, LNCC, WBC, Lymphocytes
	S1p				2. IMQ+S1p	IMQ+vehicle		HE (ET, PCNA), ICI, EE, IL-17, IL-23, LNW, LNCC, WBC, Lymphocytes, ES
Sun, 2017 (24)	FTY720	BALB/c	Female/6–8 w	62.5 mg, 5% IMQ	IMQ+FTY720	IMQ	Intraperitoneal	HE
Ji et al., 2018 (25)	FTY720	Kunming mice	Female/ 6–8 w	0.2 mg diethylstilbestrol	1. FTY720	Vehicle	Oral	Mitotic index;
	Syl930				2. Syl930	Vehicle		Mitotic index; Body weight
		Guinea pigs	Female	5% (m/v) propranolol ethanol solution containing azone and propanediol (1:3)	3. Syl930	Vehicle		HE (PS), Body weight
Shin et al., 2019 (27)	Ceranib-2/ MP-A08	C57BL/6	Male/8 w	83 mg, 5% IMQ	IMQ+Ceranib-2 IMQ+MP-A08	IMQ+EPH	Topical	PASI (erythema, scaling, skin thickness), LNCC, SpCC, SpW, IL-17A, K6, K16
	PF-543/ABC294640				IMQ+ PF-543, IMQ+ABC294640	IMQ+EPH		PASI (erythema, scaling, skin thickness), LNCC, SpCC, SpW
Qin and Zheng, 2019 (26)	FTY720	C57BL/6	Female	10 mg/d IMQ	IMQ+FTY720	IMQ+PBS	Intraperitoneal	EaT, CD3^+^ T cells
Jeon et al., 2020 (28)	SLI	BALB/c	8 w	62.5 mg, 5% IMQ	IMQ+ SLI	IMQ	Subcutaneous	HE (ET), PASI (erythema, scaling, skin thickness), EaT
Jing et al., 2020 (29)	FTY720 IMMH002	BALB/c	Female/6–8 w	50 mg, 5% IMQ	1. IMQ+FTY720	IMQ+ Vehicle	Oral	PASI (erythema, scaling, skin thickness)
					2. IMQ+IMMH002	IMQ+ Vehicle		PASI (erythema, scaling, skin thickness), CD3^+^ T cells
		Hartley guinea pigs	Female/4–6 w/250–400 g	5% propranolol in emulsifying ointment	3. FTY720	Vehicle		PS, ET, PCNA
					4. IMMH002	Vehicle		PS, ET, PCNA, IL-17
Shin et al., 2020 (30)	HWG-35D	C57BL/6	Male/8 w	83 mg, 5% IMQ	IMQ+ HWG-35D	IMQ+EPH (H_2_O)	Topical	PASI (erythema, scaling, skin thickness), LNCC, SpCC, SpW, IL-17A,/K6, K16
Okura et al., 2021 (31)	Fingolimod (FTY720)	BALB/c	Female/7–9 w	30 mg, 5% IMQ	IMQ+ Fingolimod	IMQ+PBS	Intraperitoneal	PASI, ET, CD3^+^T cells

CLA, cutaneous lymphocyte-associated antigen; DC, dendritic cells; EaT, ear thickness; EE, edema-ear; ES, ear swelling; ET, epidermal thickening; HE, hematoxylin-eosin staining; ICI, inflammatory cell influx; IMQ, imiquimod; LC, Langerhans cells; LNCC, lymph node cell count; LNW, lymph node weight; PCNA, proliferating cell nuclear antigen; PS, pathology score; S1P, sphingosine-1-phosphate; SL1, S1P lyase-specific inhibitor; SpCC, spleen cell count; SpW, spleen weight; WBC, white blood cell; w, weeks.

### Study Quality

The risk of bias of the preclinical studies was assessed using the SYRCLE risk of bias tool, and one study mentioned generating sequences by weight (26), which indicated a high risk of bias, while the other randomly assessed the outcome (29). Two studies randomly grouped and housed the mice (25, 26); in another two studies, the outcomes were assessed by independent observers blinded to the intervention (28, 29). Attrition and other biases were low risks for the nine studies because they reported the outcome indicators in detail. However, it is difficult to evaluate baseline characteristics because none of the studies provided baseline data. None of the studies described allocation concealment or blinding methods for performance or detection bias (**Supplementary Table 2**).

### Outcomes

#### Clinical Trial

Vaclavkova et al. (17) performed a randomized double-blind placebo-controlled phase II trial to evaluate the efficacy, safety, and tolerability of the S1PR agonist ponesimod for the treatment of patients with chronic plaque psoriasis. They found that after treatment with ponesimod for 16 weeks, patients who received a 20 mg dose reached at least a 75% reduction in Psoriasis Area and Severity Index (PASI75) score with a lower risk of adverse events (AEs) than those in the 40 mg and placebo groups. The main AEs were dyspnea and increased liver enzyme concentrations.

#### Preclinical Research

##### Phenotype

###### S1PR

A meta-analysis of five studies (23, 25, 26, 29, 31) evaluated the efficacy of S1PR agonists for treating psoriasis-like dermatitis. Overall, S1PR agonists improved psoriasis-like dermatitis in mice (MD: −6.80; 95% CI: −8.23, −5.38; p<0.00001) (**Supplementary Figure 1**). After intervention with agonists, ear swelling (MD: −0.06; 95% CI: −0.09 to −0.03; p<0.0001), skin erythema (MD: −0.89; 95% CI: −1.22 to −0.57; p<0.00001), thickness (MD: −0.65; 95% CI: −0.97 to −0.34; p<0.001), and total PASI scores (MD: −2.21; 95% CI: −2.88 to −1.54; p<0.00001) of psoriatic mice were significantly decreased. When analyzing the hematoxylin-eosin (HE)-stained sections of the skin lesions, the epidermal thickness (MD: −27.24; 95% CI: −48.59 to −6.16; p=0.01), pathological score (MD: −1.91; 95% CI: −2.52 to −1.30; p<0.00001), and number of proliferating cell nuclear antigen (PCNA)–positive cells (MD: −19.16; 95% CI: −36.55 to −1.76; p=0.03) in mice with psoriatic dermatitis were substantially lower (**Table 2** and **Supplementary Figure 1**).

**Table 2:** Phenotypes of S1P signal modulators for treating psoriasis.

Study or subgroup		Experiment			Control			Mean difference [95% CI]	I²	P value
		Mean	SD	Total	Mean	SD	Total			
**1. S1PR agonists**										
1.1 PASI scores	Jing et al., 2020-1 (29)	5.26	1.14	10	7.26	1.14	10	-2.00 [-3.00, -1.00]		
	Jing et al., 2020-2 (29)	5.02	1.28	10	7.26	1.28	10	-2.24 [-3.36, -1.12]		
	Okura et al., 2021 (31)	4.95	1.25	5	7.60	1.25	5	-2.65 [-4.20, -1.10]		
	Subtotal (95% CI)			25			25	-2.21 [-2.88, -1.54]	0%	<0.00001
1.2 PASI-Erythema	Jing et al., 2020-1 (29)	1.21	0.45	10	1.99	0.45	10	-0.78 [-1.17, -0.39]		
	Jing et al., 2020-2 (29)	0.87	0.64	10	1.99	0.64	10	-1.12 [-1.68, -0.56]		
	Subtotal (95% CI)			20			20	-0.89 [-1.22, -0.57]	0%	<0.00001
1.3 PASI-Thickness	Jing et al., 2020-1 (29)	2.17	0.50	10	2.82	0.50	10	-0.65 [-1.09, -0.21]		
	Jing et al., 2020-2 (29)	2.16	0.51	10	2.82	0.51	10	-0.66 [-1.11, -0.21]		
	Subtotal (95% CI)			20			20	-0.65 [-0.97, -0.34]	0%	<0.0001
1.4 Ear swelling	Qin and Zheng, 2019 (26)	0.30	0.20	6	0.34	0.20	6	-0.04 [-0.27, 0.19]		
	Schaper et al., 2013-2 (23)	0.09	0.02	6	0.15	0.03	6	-0.06 [-0.09, -0.03]		
	Subtotal (95% CI)			12			12	-0.06 [-0.09, -0.03]	0%	<0.0001
1.5 HE pathology score	Ming et al., 2018-3 (25)	0.31	0.20	5	2.82	0.53	5	-2.51 [-3.01, -2.01]		
	Jing et al., 2020-3 (29)	0.89	0.34	8	2.26	0.46	8	-1.37 [-1.77, -0.97]		
	Jing et al., 2020-4 (29)	0.37	0.32	8	2.26	0.46	8	-1.89 [-2.28, -1.50]		
	Subtotal (95% CI)			21			21	-1.91 [-2.52, -1.30]	84%	<0.00001
1.6 HE Epidermal thickness	Jing et al., 2020-3 (29)	80.30	8.12	8	108.27	7.22	8	-27.97 [-35.50, -20.44]		
	Jing et al., 2020-4 (29)	67.67	6.77	8	108.27	7.22	8	-40.60 [-47.46, -33.74]		
	Okura et al., 2021 (31)	138.14	28.46	5	228.87	28.46	5	-90.73 [-126.01, -55.45]		
	Schaper et al., 2013-1 (23)	62.22	4.68	6	58.25	5.85	6	3.97 [-2.02, 9.96]		
	Schaper et al., 2013-2 (23)	53.57	9.36	6	58.25	5.85	6	-4.68[-13.51, 4.15]		
	Subtotal (95% CI)			33			33	-27.42 [-48.69, -6.16]	97%	0.01
1.7 HE PCNA positive cells	Jing et al., 2020-3 (29)	56.25	1.47	8	79.41	1.10	8	-23.16 [-24.43, -21.89]		
	Jing et al., 2020-4 (29)	33.46	2.57	8	79.41	1.10	8	-45.95 [-47.89, -44.01]		
	Schaper et al., 2013-1 (23)	23.12	1.70	6	24.68	4.68	6	-1.56 [-5.54, 2.42]		
	Schaper et al., 2013-2 (23)	19.09	3.12	6	24.68	4.68	6	-5.59 [-10.09, -1.09]		
	Subtotal (95% CI)			28			28	-19.16 [-36.55, -1.76]	100%	0.03
	Total (95% CI)			159			159	-6.80 [-8.23, -5.38]	99%	<0.00001
**2. SPHK2 inhibitor**										
2.1 PASI scores	Shin et al., 2019 (27)	1.86	0.55	5	3.03	0.55	5	-1.17 [-1.85, -0.49]		
	Shin et al., 2020 (30)	5.13	0.74	5	7.50	0.74	5	-2.37 [-3.29, -1.45]		
	Subtotal (95% CI)			10			10	-1.73 [-2.90, -0.56]	76%	0.004
2.2 PASI-Erythema	Shin et al., 2019 (27)	2.03	0.28	5	2.93	0.28	5	-0.90 [-1.25, -0.55]		
	Shin et al., 2020 (30)	2.25	0.23	5	2.99	0.23	5	-0.74 [-1.03, -0.45]		
	Subtotal (95% CI)			10			10	-0.80 [-1.02, -0.58]	0%	<0.00001
2.3 PASI-Thickness	Shin et al., 2019 (27)	2.30	0.37	5	3.08	0.37	5	-0.78 [-1.24, -0.32]		
	Shin et al., 2020 (30)	1.81	0.32	5	2.83	0.32	5	-1.02 [-1.42, -0.62]		
	Subtotal (95% CI)			10			10	-0.92 [-1.22, -0.62]	0%	<0.00001
2.4 PASI-Scale	Shin et al., 2019 (27)	1.77	0.64	5	3.12	0.64	5	-1.35 [-2.14, -0.56]		
	Shin et al., 2020 (30)	1.67	0.13	5	2.08	0.13	5	-0.41 [-0.57, -0.25]		
	Subtotal (95% CI)			10			10	-0.80 [-1.70, 0.11]	81%	0.09
	Total (95% CI)			40			40	-0.95 [-1.26, -0.65]	79%	<0.00001

CI, confidence interval; HE, hematoxylin and eosin; PASI, Psoriasis Area and Severity Index; PCNA, proliferating cell nuclear antigen; S1PR, sphingosine-1-phosphate receptor; SPHK2, sphingosine kinase 2.

##### SPHK1/2

Shin et al. (27) reported that both Ceranib-2 and MP-A08 improved the severity of psoriasis-like skin lesions with reduced PASI scores, erythema, scaling, and epidermal thickness and upregulated cellular proliferation. In particular, the effect of the topical application of a specific SPHK2 inhibitor on imiquimod (IMQ)–induced skin disease was more significant than that of the SPHK1 inhibitor (27, 30). We found that the SPHK2 inhibitor significantly reduced the skin erythema (MD: −0.80; 95% CI: −1.02 to −0.58; p<0.00001), epidermal thickness (MD: −0.92; 95% CI: −1.22 to −0.62; p<0.00001), and total PASI scores (MD: −1.73; 95% CI: −2.90 to −0.56; p=0.004), while skin scales (MD: −0.80; 95% CI: −1.70 to 0.11; p=0.09) were not significantly changed after treatment with a topical SHPK2 inhibitor (**Table 2** and **Supplementary Figure 2**).

##### SL1

Jeon et al. reported that the S1P lyase inhibitor SLI alleviated psoriatic lesions and reduced epidermal thickness and mean PASI scores (28).

#### Mechanisms

##### S1PR

We first analyzed the inflammatory state of ear lesions in psoriasis-like mice, and the meta-analysis showed that IL-23 mRNA levels in the ear lesions of psoriatic mice were significantly decreased (MD: −4.35; 95% CI: −7.64 to −1.06; p=0.009, **Supplementary Figure 3**) in the S1PR agonist group, while the ear inflammatory cell influx (**Supplementary Figure 4**) and IL-17 mRNA production did not change markedly (p>0.05, **Supplementary Figure 5**). In addition, after the S1PR agonist intervention, lymph node weight was reduced (MD: −2.49; 95% CI: −4.05 to −0.93; p=0.002; **Supplementary Figure 6**), while the cell count did not change significantly (p=0.10; **Supplementary Figure 7**). Moreover, there was almost no change in the number of lymphocytes in the blood after S1PR agonist treatment (p=0.10; **Supplementary Figure 8**). However, in the peripheral tissues, the percentage of CD3^+^ T lymphocytes (including in the ears, blood, and spleen) decreased significantly after treatment with S1PR agonists (MD: −10.35; 95% CI: −15.19 to −5.51; p<0.0001; **Supplementary Figure 9**). We also found that the number of white blood cells (WBCs) was markedly reduced (MD: −1.67; 95% CI: −3.20 to −0.15; p=0.03; **Supplementary Figure 10**) relative to the control group. The details are listed in **Table 3**.

**Table 3:** Mechanisms of S1P signal modulators in treating psoriasis.

Study or subgroup	Experiment			Control			Mean difference[95%CI]	I²	P value
	Mean	SD	Total	Mean	SD	Total			
**1. S1PR agonists**									
** 1.1 Ear lesions**									
*** 1.1.1 Ear-Inflammatory cell influx***									
Schaper et al., 2013-1 (23)	2.3	0.6	6	2.4	0.6	6	-0.10 [-0.78, 0.58]		
Schaper et al., 2013-2 (23)	1.7	0.7	6	2.4	0.6	6	-0.70 [-1.44, 0.04]		
Total (95%CI)			12			12	-0.38 [-0.97, 0.20]	27%	0.20
*** 1.1.2 IL-17***									
Schaper et al., 2013-1 (23)	21	6	3	48	18	3	-27.00 [-48.47, -5.53]		
Schaper et al., 2013-2 (23)	59	20	3	48	18	3	11.00 [-19.45, 41.45]		
Total (95%CI)			6			6	-9.60 [-46.70, 27.51]	75%	0.61
*** 1.1.3 IL-23***									
Schaper et al., 2013-1 (23)	7	6	6	10	4	6	-3.00 [-8.77, 2.77]		
Schaper et al., 2013-2 (23)	5	3	6	10	4	6	-5.00 [-9.00, -1.00]		
Total (95%CI)			12			12	-4.35 [-7.64, -1.06]	0%	0.009
** 1.2 White blood cells**									
Schaper et al., 2013-1 (23)	5.8	1.7	6	7.7	1.3	6	-1.90 [-3.61, -0.19]		
Schaper et al., 2013-2 (23)	6.9	4.0	6	7.7	1.3	6	-0.80 [-4.17, 2.57]		
Total (95%CI)			12			12	-1.67 [-3.20, -0.15]	0%	0.03
** 1.3 Lymph node**									
*** 1.3.1 Lymph node weight (mg)***									
Schaper et al., 2013-1 (23)	4.2	0.8	6	7.0	2.5	6	-2.80 [-4.90, -0.70]		
Schaper et al., 2013-2 (23)	4.9	1.5	6	7.0	2.5	6	-2.10 [-4.43, 0.23]		
Total (95%CI)			12			12	-2.49 [-4.05, -0.93]	0%	0.002
1.3.2 Lymph node cell count (*10^6^)									
Schaper et al., 2013-1 (23)	6.4	1.7	6	8.6	3.0	6	-2.20 [-4.96, 0.56]		
Schaper et al., 2013-2 (23)	7.4	3.3	6	8.6	3.0	6	-1.20 [-4.77, 2.37]		
Total (95%CI)			12			12	-1.83 [-4.01, 0.36]	0%	0.10
** 1.4 Lymphocytes in blood**									
Schaper et al., 2013-1 (23)	2.0	0.9	6	3.8	0.7	6	-1.80 [-2.71, -0.89]		
Schaper et al., 2013-2 (23)	3.5	1.9	6	3.8	0.7	6	-0.30 [-1.92, 1.32]		
Total (95%CI)			12			12	-1.21 [-2.64, 0.23]	60%	0.10
** 1.5 CD3^+^T cells in peripheral tissues**									
Qin and Zheng, 2019 (26)	6.63	0.54	6	14.34	1.38	6	-7.71 [-8.90, -6.52]		
Jin Jing et al., 2020-2 (29)	3.75	0.92	10	9.76	1.23	10	-17.84 [-20.33, -15.35]		
Jin Jing et al., 2020-2-1 (29)	24.47	2.93	10	42.31	2.75	10	-6.01 [-6.96, -5.06]		
Total (95%CI)			26			26	-10.35 [-15.19, -5.51]	97%	<0.0001
**2. SPHK2 Inhibitor**									
** 2.1 Spleen weight (g)**									
Shin et al., 2019 (27)	0.12	0.01	5	0.14	0.01	5	-0.02 [-0.03, -0.01]		
Shin et al., 2020 (30)	0.14	0.02	5	0.19	0.02	5	-0.05 [-0.07, -0.03]		
Total (95%CI)			10			10	-0.03 [-0.06, -0.00]	78%	0.03
2.2 Spleen cell number (*10^6^)									
Shin et al., 2019 (27)	90.83	14.16	5	120.88	14.16	5	-30.05 [-47.60, -12.50]		
Shin et al., 2020 (30)	52.60	10.41	5	74.68	10.41	5	-22.08 [-34.98, -9.18]		
Total (95%CI)			10			10	-24.88 [-35.27, -14.48]	0%	<0.00001
2.3 Lymph node cell number (*10^5^)									
Shin et al., 2019 (27)	368.16	70.41	5	517.57	70.41	5	-149.41 [-236.69, -62.13]		
Shin et al., 2020 (30)	2.43	2.36	5	7.43	2.36	5	-5.00 [-7.93, -2.07]		
Total (95%CI)			10			10	-5.16 [-8.09, -2.24]	90%	0.33

##### SPHK1/2

Shin et al. (27) reported that, after treatment with Ceranib-2 or MP-A08, IL-17A, IL-17F, and TNF-α levels were significantly decreased (p<0.05) compared with those in IMQ-induced skin inflammation. Levels of keratinocyte hyperproliferation markers K6 and K16 were also significantly suppressed (p<0.05). A meta-analysis showed that SPHK2 inhibitors reduced spleen weight (MD: −0.03; 95% CI: −0.06 to −0.00; p=0.03; **Supplementary Figure 11**) and spleen cell numbers (MD: −24.88; 95% CI: −35.27 to −14.48; p<0.00001; **Supplementary Figure 12**). However, the number of lymph nodes did not significantly decrease (p>0.05; **Supplementary Figure 13**). The details are listed in **Table 3**.

## Discussion

**Figure 2:**
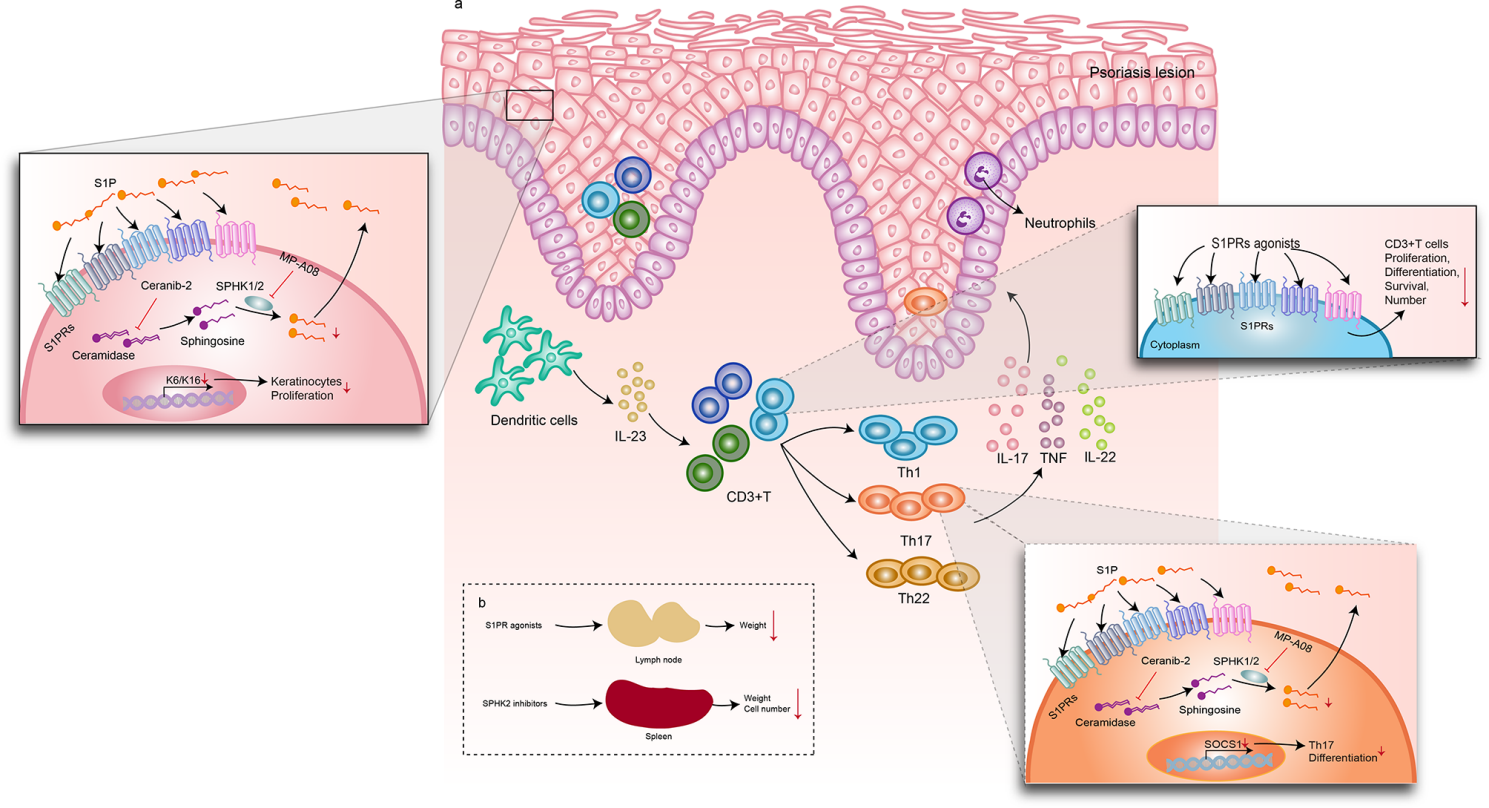
Mechanisms of S1P signaling–related drugs in psoriasis-like dermatitis. S1PR agonists, including S1P, FTY720 (fingolimod), SYl930, and IMMH002. **(A)** After intervention with S1PR agonists, the lymph node weight decreased. Spleen weight and cell number were downregulated by SPHK2 inhibitors. The DC/T cells/KC axis plays an important role in the pathogenesis of psoriasis. In psoriatic plaque lesions, DCs release IL-23 and IL-12 to activate Th1, TH17, Th22, and other lymphocytes to produce abundant inflammatory factors, such as IL-17, IFN-γ, TNF-α, and IL-22. These cytokines trigger excessive keratinocyte proliferation and amplify psoriasis-related inflammation. When S1PR agonists were used, the numbers of WBCs and CD3^+^ T cells decreased and IL-23 expression decreased. A ceramidase inhibitor, Ceranib-2, and an SPHK1/2 inhibitor, MP-A08, suppressed S1P synthesis, resulting in decreased levels of IL-17A, IL-17F, and TNF-α in Th17 cells. In keratinocytes, Ceranib-2 and MP-A08 reduced the markers of keratinocyte hyperproliferation and keratins K6 and K16. Moreover, MP-A08 significantly suppressed SOCS1expression, which reduced the differentiation of Th17 cells. **(A)** Effect of S1P and its signal modulators on psoriatic skin lesions; **(B)** Effect of S1P and its signal modulators on draining lymph nodes and spleen. DC, dendritic cells; KC, keratinocytes; S1P, sphingosine-1-phosphate; S1PR, sphingosine-1-phosphate receptor; SPHK, sphingosine kinase; Th1, T helper 1; Th17, T helper 17; Th22, T helper 22.

To our knowledge, this is the first systematic review and meta-analysis of clinical and preclinical animal studies to evaluate the efficacy and mechanism of S1P and its signal modulators for treating psoriasis and psoriasis-like mice (**Figure 2**). This study mainly included literature of clinical and preclinical research. In the preclinical research section, we subdivided the basic research of S1P and its modulators for psoriasis into phenotypes and mechanisms for the meta-analysis. In this study, we classified intervention drugs into S1PR agonists, SPHK inhibitors, and S1P lyases according to their different roles in the SIP signaling pathway. The S1PR agonists in this systematic review included S1P, FTY720, Syl930, and IMMH002. FTY720, also known as fingolimod, is an analog of S1P derived from the structure-modified extract of *Cordyceps sinensis*. It is the first S1PR modulator approved by the US Food and Drug Administration for the treatment of relapsing multiple sclerosis (32). FTY720 binds to four of five S1PRs (S1PR1, S1PR3, S1PR4, S1PR5) to reduce the number of lymphocytes in the blood and inhibit the infiltration of inflammatory cells (13). Syl930 and IMMH002, developed by a Chinese team, are selective S1PR1 modulators, such as FTY720, that regulate the distribution of lymphocytes by inducing lymphocyte homing to treat psoriasis (29).

In patients with psoriasis, the serum concentration of S1P increases (16). Shin et al. used Ceranib-2, a ceramidase inhibitor, and MP-A08, an SPHK1/2 inhibitor, to prevent S1P synthesis. They found that topical inhibition of S1P production improves inflammation in psoriasis-like mice, possibly by blocking Th17 cell differentiation (27). In particular, SPHK2 inhibitors such as ABC294640 and HWG-35D have the greatest inhibitory effect on S1P synthesis (27, 30). Several studies have reported that S1P induces keratinocyte differentiation (33, 34). Jeon et al. used an SLI to inhibit S1P lyase activity in human keratinocytes and observed the same phenomenon. In addition, the severity of psoriasis-like dermatitis in mice is alleviated (28). Our findings point to the improvement of psoriasis by inhibiting S1P expression or synthesis. Only clinical study to date has reported on the clinical efficacy and safety of S1PR. It is recommended that 20 mg ponesimod orally can achieve good efficacy with fewer adverse reactions. However, a recent meta-analysis reported that patients receiving S1P modulators were at an increased risk of infection and transient cardiovascular events (35). As these modulators can be considered potential drugs for the treatment of psoriasis, future large-scale clinical research on different dosage forms and chemical modifications to reduce toxicity can be pursued. Additionally, S1P signaling plays a complex role in different cells, and further studies are needed to explain the mechanism underlying psoriasis.

This systematic review has some limitations. First, the methodological quality of the enrolled animal studies was low, and most had a high risk of selection bias. Second, because there are few studies on the mechanism of S1P and its signal modulators for psoriasis, the number of articles included was limited, which reduced our ability to reach a definitive conclusion. Third, the PASI score, the gold standard method for evaluating improvement in psoriasis, was not used in all studies.

## Conclusion

Our findings indicate that psoriasis can be treated by blocking S1P activity by competitively binding to S1PR and inhibiting S1P synthase activity (SPHK2 inhibitor) using a mechanism that is related to decreased immune responses and inflammatory factor levels. These drugs improve psoriatic dermatitis by decreasing the immune response, such as by reducing the number of immune cells including lymphocytes, and downregulating the secretion of inflammatory factors.

## Data Availability Statement

The original contributions presented in the study are included in the article/**Supplementary Material**. Further inquiries can be directed to the corresponding authors.

## Author Contributions

XL and BL proposed and designed the study. They also obtained funding support. JW, SZ, S-tC, H-jL, and X-yS retrieved and selected the data. Y-qZ and M-ZJ extracted the data. YinL and YR assessed the quality of all studies. LL, YiL, DY, and Y-qZ performed the statistical analysis of all data. LL and JW drafted the manuscript, and XL revised it.

## Funding

This work was supported by the National Natural Science Foundation of China (grant no. 81874470 and 82074427), the National Key Research and Development Program of China (grant no. 2018YFC1705301), the National Natural Science Foundation of Shanghai (grant no. 19ZR1458700), and Shanghai Pujiang Program (grant no. 2020PJD067).

## Conflict of Interest

The authors declare that the research was conducted in the absence of any commercial or financial relationships that could be construed as a potential conflict of interest.

## Publisher’s Note

All claims expressed in this article are solely those of the authors and do not necessarily represent those of their affiliated organizations, or those of the publisher, the editors and the reviewers. Any product that may be evaluated in this article, or claim that may be made by its manufacturer, is not guaranteed or endorsed by the publisher.

## Supplementary Material

Supplementary Figure 1 Meta-analysis of the phenotype of psoriasis-like dermatitis after treatment with an S1PR agonist. 95% CI, 95% confidence interval; S1PR, sphingosine-1-phosphate receptor

Supplementary Figure 2 Meta-analysis of phenotype in psoriasis-like dermatitis after treatment with an SPHK2 inhibitor. 95% CI, 95% confidence interval; SPHK2, sphingosine kinase 2.

Supplementary Figure 3 Meta-analysis of IL-23 levels in psoriasis-like dermatitis, after treatment with an S1PR agonist. 95% CI, 95% confidence interval; IL-23, interleukin-23; S1PR, sphingosine-1-phosphate receptor.

Supplementary Figure 4 Meta-analysis of IL-17 levels in psoriasis-like dermatitis after treatment with an S1PR agonist. 95% CI, 95% confidence interval; IL-17, interleukin-17; S1PR, sphingosine-1-phosphate receptor.

Supplementary Figure 5 Meta-analysis of ear inflammatory cell influx in psoriasis-like dermatitis after treatment with an S1PR agonist. 95% CI, 95% confidence interval; S1PR, sphingosine-1-phosphate receptor

Supplementary Figure 6 Meta-analysis of lymph node weight in psoriasis-like dermatitis after treatment with an S1PR agonist. 95% CI, 95% confidence interval; S1PR, sphingosine-1-phosphate receptor.

Supplementary Figure 7 Meta-analysis of lymph node cell counts in psoriasis-like dermatitis after treatment with an S1PR agonist. 95% CI, 95% confidence interval; S1PR, sphingosine-1-phosphate receptor.

Supplementary Figure 8 Meta-analysis of lymphocytes in blood in psoriasis-like dermatitis after treatment with an S1PR agonist. 95% CI, 95% confidence interval; S1PR, sphingosine-1-phosphate receptor.

Supplementary Figure 9 Meta-analysis of CD3^+^ T cells in psoriasis-like dermatitis after treatment with an S1PR agonist. 95% CI, 95% confidence interval; S1PR, sphingosine-1-phosphate receptor.

Supplementary Figure 10 Meta-analysis of white blood cells in psoriasis-like dermatitis after treatment with an S1PR agonist. 95% CI, 95% confidence interval; S1PR, sphingosine-1-phosphate receptor.

Supplementary Figure 11 Meta-analysis of spleen weight in psoriasis-like dermatitis after treatment with an SPHK2 inhibitor. 95% CI, 95% confidence interval; SPHK2, sphingosine kinase 2.

Supplementary Figure 12 Meta-analysis of spleen cell numbers in psoriasis-like dermatitis after treatment with an SPHK2 inhibitor. 95% CI, 95% confidence interval; SPHK2, sphingosine kinase 2.

Supplementary Figure 13 Meta-analysis of lymph node cell numbers in psoriasis-like dermatitis after treatment with an SPHK2 inhibitor. 95% CI, 95% confidence interval; SPHK2, sphingosine kinase 2.
