# Supplementary figures and images for: Sphingosine-1-Phosphate and Its Signal Modulators Alleviate Psoriasis-Like Dermatitis: Preclinical and Clinical Evidence and Possible Mechanisms
Source: Front Immunol. 2021 Dec 21;12:759276. doi: 10.3389/fimmu.2021.759276 (PMC8724303; doi:10.3389/fimmu.2021.759276)

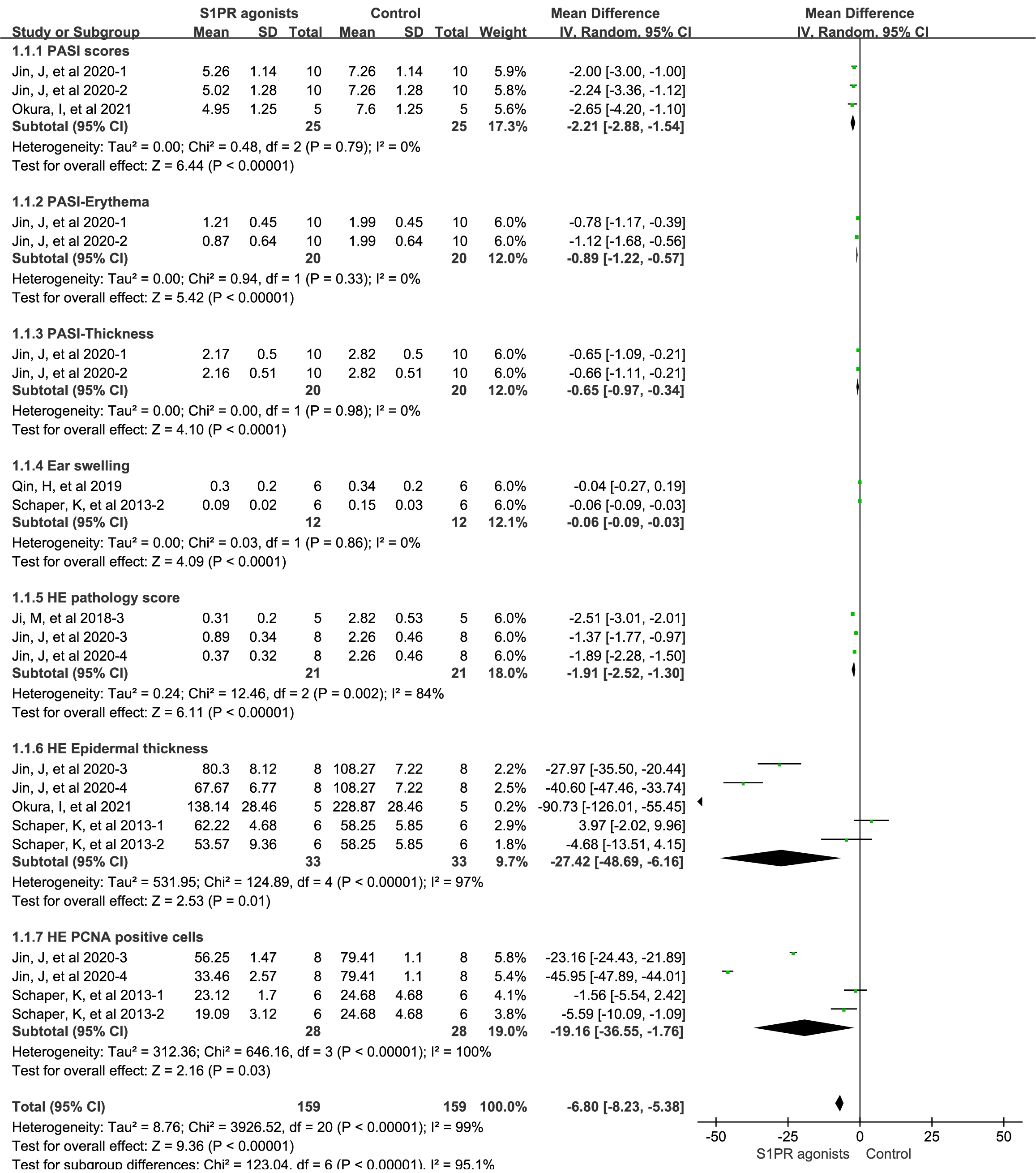

Supplement: Supplementary Figure 1 — Meta-analysis of the phenotype of psoriasis-like dermatitis after treatment with an S1PR agonist. 95% CI, 95% confidence interval; S1PR, sphingosine-1-phosphate receptor [file Image_1.tif]

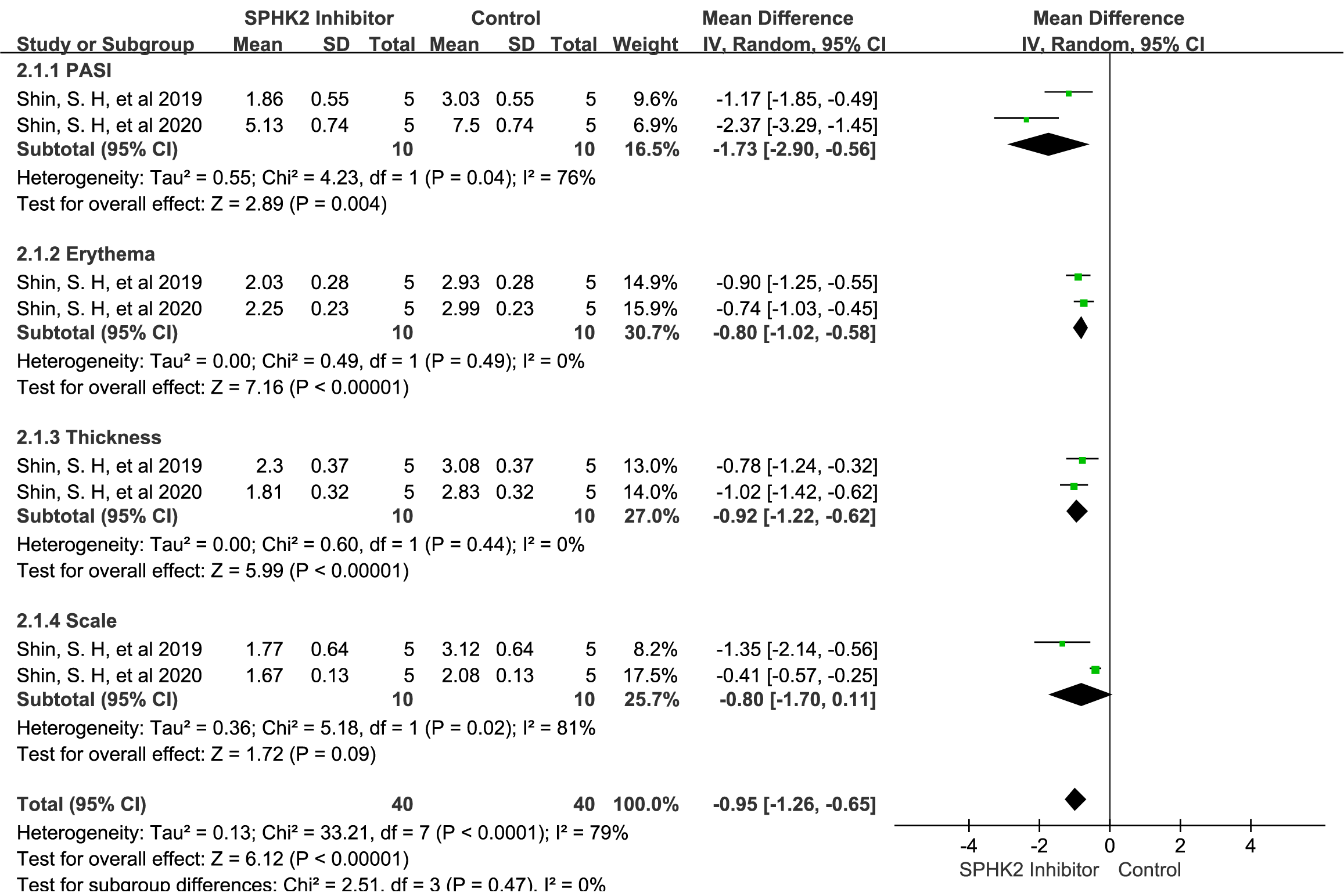

Supplement: Supplementary Figure 2 — Meta-analysis of phenotype in psoriasis-like dermatitis after treatment with an SPHK2 inhibitor. 95% CI, 95% confidence interval; SPHK2, sphingosine kinase 2. [file Image_2.tif]

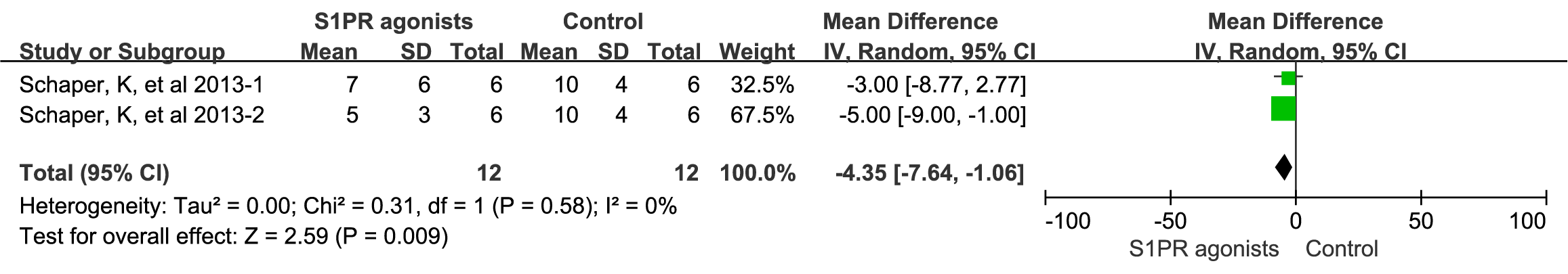

Supplement: Supplementary Figure 3 — Meta-analysis of IL-23 levels in psoriasis-like dermatitis, after treatment with an S1PR agonist. 95% CI, 95% confidence interval; IL-23, interleukin-23; S1PR, sphingosine-1-phosphate receptor. [file Image_3.tif]

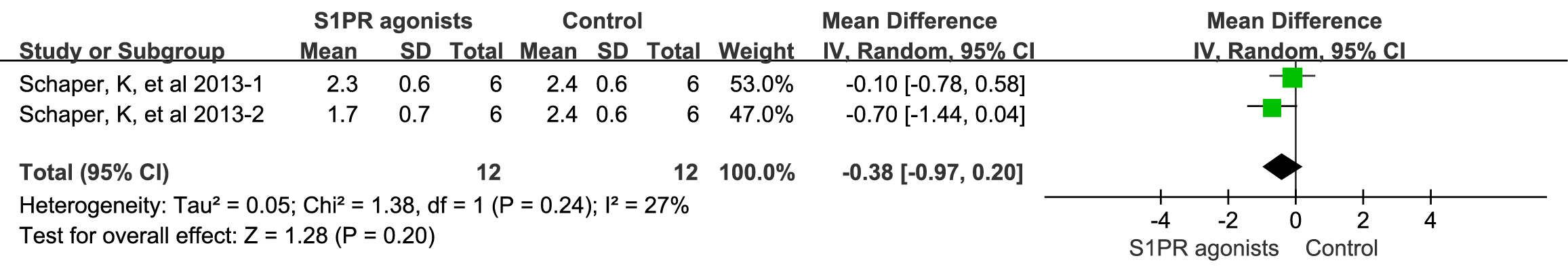

Supplement: Supplementary Figure 4 — Meta-analysis of IL-17 levels in psoriasis-like dermatitis after treatment with an S1PR agonist. 95% CI, 95% confidence interval; IL-17, interleukin-17; S1PR, sphingosine-1-phosphate receptor. [file Image_4.tif]

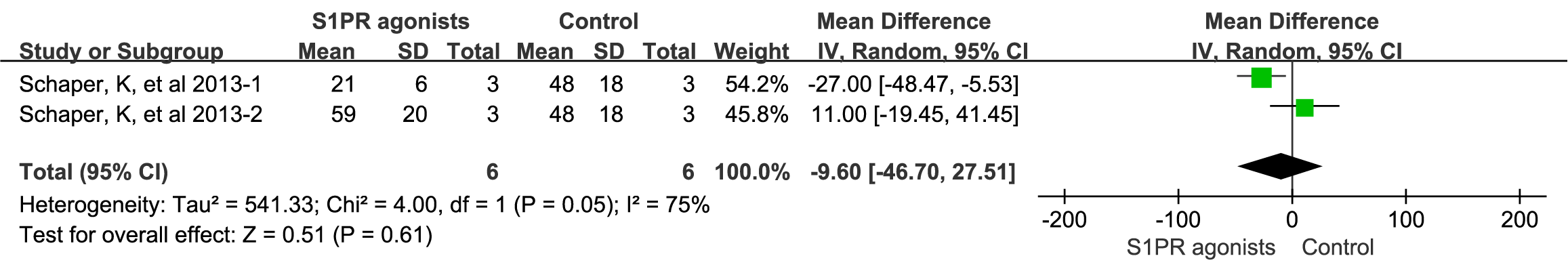

Supplement: Supplementary Figure 5 — Meta-analysis of ear inflammatory cell influx in psoriasis-like dermatitis after treatment with an S1PR agonist. 95% CI, 95% confidence interval; S1PR, sphingosine-1-phosphate receptor [file Image_5.tif]

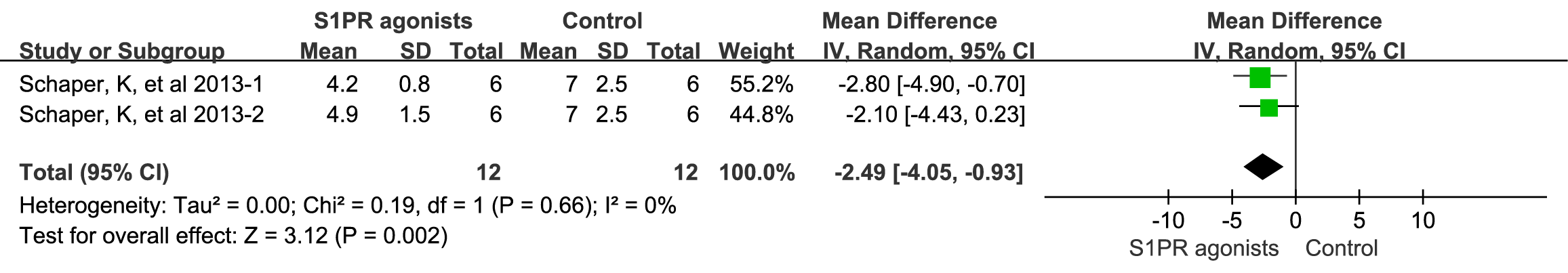

Supplement: Supplementary Figure 6 — Meta-analysis of lymph node weight in psoriasis-like dermatitis after treatment with an S1PR agonist. 95% CI, 95% confidence interval; S1PR, sphingosine-1-phosphate receptor. [file Image_6.tif]

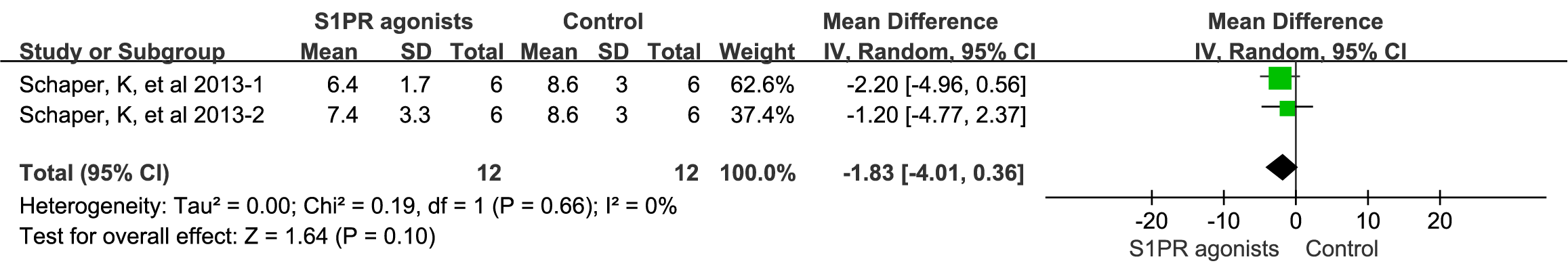

Supplement: Supplementary Figure 7 — Meta-analysis of lymph node cell counts in psoriasis-like dermatitis after treatment with an S1PR agonist. 95% CI, 95% confidence interval; S1PR, sphingosine-1-phosphate receptor. [file Image_7.tif]

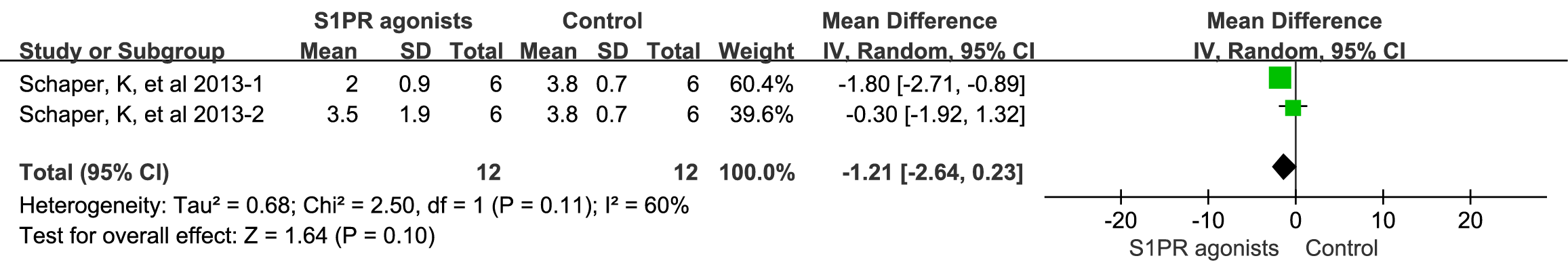

Supplement: Supplementary Figure 8 — Meta-analysis of lymphocytes in blood in psoriasis-like dermatitis after treatment with an S1PR agonist. 95% CI, 95% confidence interval; S1PR, sphingosine-1-phosphate receptor. [file Image_8.tif]

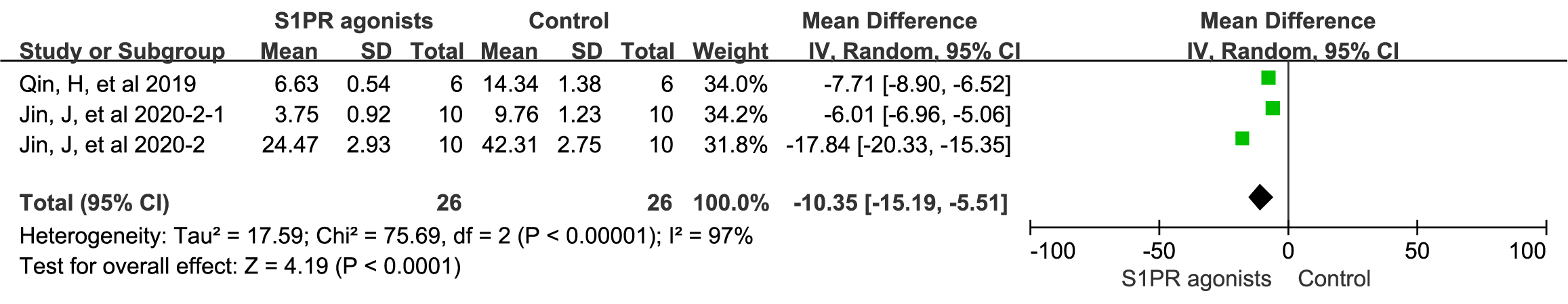

Supplement: Supplementary Figure 9 — Meta-analysis of CD3+ T cells in psoriasis-like dermatitis after treatment with an S1PR agonist. 95% CI, 95% confidence interval; S1PR, sphingosine-1-phosphate receptor. [file Image_9.tif]

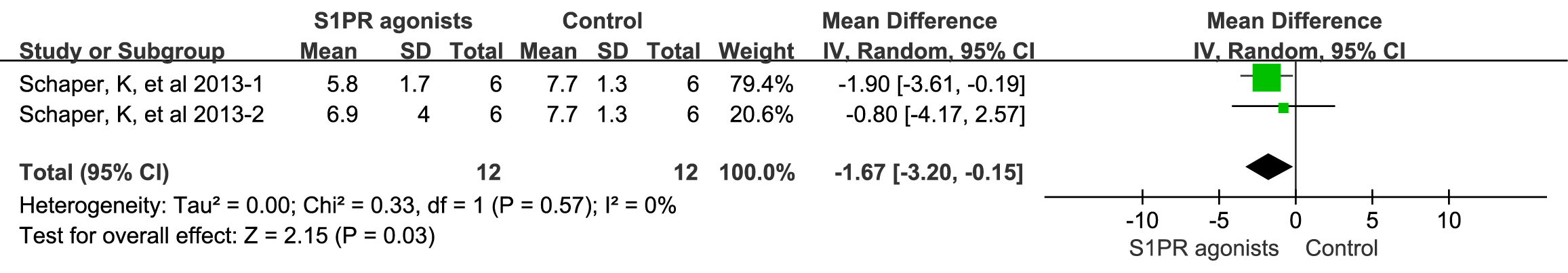

Supplement: Supplementary Figure 10 — Meta-analysis of white blood cells in psoriasis-like dermatitis after treatment with an S1PR agonist. 95% CI, 95% confidence interval; S1PR, sphingosine-1-phosphate receptor. [file Image_10.tif]

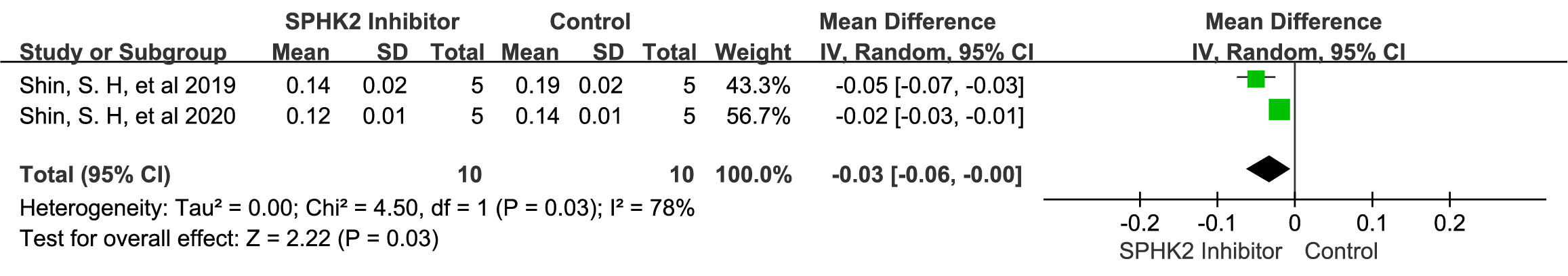

Supplement: Supplementary Figure 11 — Meta-analysis of spleen weight in psoriasis-like dermatitis after treatment with an SPHK2 inhibitor. 95% CI, 95% confidence interval; SPHK2, sphingosine kinase 2. [file Image_11.tif]

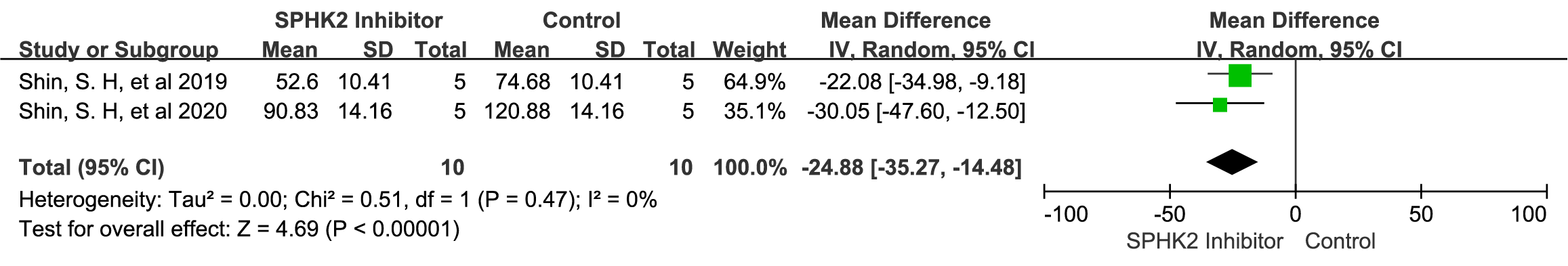

Supplement: Supplementary Figure 12 — Meta-analysis of spleen cell numbers in psoriasis-like dermatitis after treatment with an SPHK2 inhibitor. 95% CI, 95% confidence interval; SPHK2, sphingosine kinase 2. [file Image_12.tif]

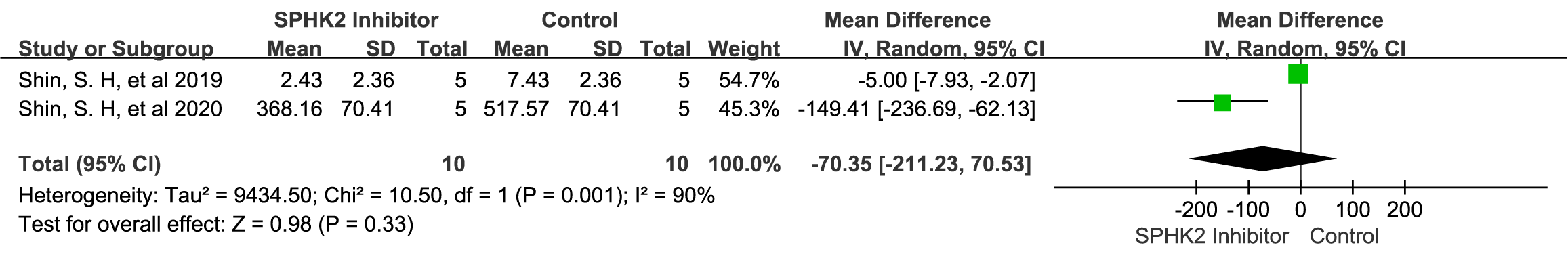

Supplement: Supplementary Figure 13 — Meta-analysis of lymph node cell numbers in psoriasis-like dermatitis after treatment with an SPHK2 inhibitor. 95% CI, 95% confidence interval; SPHK2, sphingosine kinase 2. [file Image_13.tif]
