# Supplementary material for: Sphingosine-1-Phosphate and Its Signal Modulators Alleviate Psoriasis-Like Dermatitis: Preclinical and Clinical Evidence and Possible Mechanisms
Source: Front Immunol. 2021 Dec 21;12:759276. doi: 10.3389/fimmu.2021.759276 (PMC8724303; doi:10.3389/fimmu.2021.759276)
Supplement: Supplementary file 14 [file Table_1.docx]

| **Supplementary table 1. Characteristics of the included clinical trial** | | | | | | | | | | |
| --- | --- | --- | --- | --- | --- | --- | --- | --- | --- | --- |
| ***Study***  ***(author/year)*** | ***Drug*** | ***Design*** | ***Sample size*** | ***Psoriasis type*** | ***Age*** | | ***Interventions*** | | ***Course of treatment*** | ***Outcomes*** |
|  |  |  |  |  | ***I*** | ***C*** | ***I*** | ***C*** |  |  |
| Vaclavkova, A, *et al*. 2014 | Ponesimod | Randomization number, double-blind, multi-center, phase trial | Ia. 126  Ib. 133  C, 67 | Moderate-to-severe plaque psoriasis | Ia. 39.4±10.3  Ib. 40.7±10.0 | 38.8±9.7 | Ia, 0–7 d, 10 mg;  ≥8 d 20 mg;  Ib, 0–14 d, 10 mg;  ≥15 d 40 mg | 20 mg/40 mg | Induction period, 0–16 w;  Maintenance period 16–28 w | PASI, PGA, AE, SAE, PGPA, DLQI, SF-36 |

AE, adverse events; C, control; DLQI, Dermatology Life Quality Index; I/Ia/Ib, Intervention; w, weeks; PASI, Psoriasis Area and Severity Index; PGA, static physician global assessment; PGPA, patient global psoriasis assessment; SAE, severe adverse events; SF-36, Medical Outcomes Study 36-item short-form health survey.
