# Supplementary material for: Sphingosine-1-Phosphate and Its Signal Modulators Alleviate Psoriasis-Like Dermatitis: Preclinical and Clinical Evidence and Possible Mechanisms
Source: Front Immunol. 2021 Dec 21;12:759276. doi: 10.3389/fimmu.2021.759276 (PMC8724303; doi:10.3389/fimmu.2021.759276)
Supplement: Supplementary file 15 [file Table_2.docx]

| **Supplementary table 2. SYRCLE’s risk of bias tool for preclinical studies** | | | | | | | | | | |
| --- | --- | --- | --- | --- | --- | --- | --- | --- | --- | --- |
|  | ***Selection bias*** | | | ***Performance bias*** | | ***Detection bias*** | | ***Attrition bias*** | ***Reporting bias*** | ***Other bias*** |
|  | ***Sequence generation*** | ***Baseline characteristics*** | ***Allocation concealment*** | ***Random housing*** | ***Blinding*** | ***Random outcome assessment*** | ***Blinding*** | ***Incomplete outcome data*** | ***Selective outcome reporting*** |  |
| Schaper, K, *et al* 2013 | ? | ? | No | ? | No | ? | ? | Yes | Yes | Yes |
| Sun, YQ 2017 | ? | ? | No | ? | No | ? | ? | Yes | Yes | Yes |
| Ji, M, *et al*  2018 | ? | ? | No | Yes | No | ? | ? | Yes | Yes | Yes |
| Shin, S. H, *et al* 2019 | ? | ? | No | ? | No | ? | ? | Yes | Yes | Yes |
| Qin, H, *et al* 2019 | No | ? | ? | Yes | ? | ? | ? | Yes | Yes | Yes |
| Jeon, S, *et al* 2020 | ? | ? | No | ? | No | ? | Yes | Yes | Yes | Yes |
| Jin, J, *et al*  2020 | ? | ? | No | ? | No | Yes | Yes | Yes | Yes | Yes |
| Shin, S. H, *et al* 2020 | ? | ? | ? | ? | ？ | ? | ? | Yes | Yes | Yes |
| Okura, I, *et al* 2021 | ? | ? | ? | ? | ？ | ? | ？ | Yes | Yes | Yes |

“Yes”, indicate low risk of bias; “No” indicate high risk of bias; “?” indicate unclear risk of bias.
